# Impact of diabetes mellitus on risk of major complications after hip fracture: a systematic review and meta-analysis

**DOI:** 10.1186/s13098-022-00821-0

**Published:** 2022-04-12

**Authors:** Qiu Shen, Yunping Ma

**Affiliations:** Department of Orthopaedics, Huzhou Central Hospital, Affiliated Central Hospital Huzhou University, 1558 Sanhuan North Road, Huzhou, 313000 Zhejiang China

**Keywords:** Hip fracture, Complications, Mortality, Hyperglycemia

## Abstract

**Background:**

The impact of diabetes mellitus (DM) on adverse outcomes in hip fracture patients is unclear. Furthermore, no review has synthesized evidence on this subject. Therefore, the current study was designed to answer the following research question: Does DM increase the risk of mortality and major systemic complications in patients with hip fractures?

**Methods:**

PubMed, Embase, and Google Scholar were searched from 1st January 2000 to 1st August 2021 for studies comparing DM and non-DM patients with hip fractures. Outcomes of interest were pooled using risk ratios (RR). The study was registered on PROSPERO (CRD42021268525).

**Results:**

Sixteen studies were included. Meta-analysis revealed a statistically significant increased risk of mortality in diabetics as compared to non-diabetics after 1 year (RR: 1.24 95% CI 1.08, 1.43 I^2^ = 62% p = 0.003). Pooled analysis of eight studies reporting adjusted mortality outcomes also demonstrated similar results (RR: 1.17 95% CI 1.09, 1.25 I^2^ = 74% p < 0.0001). We noted a statistically significant increase in the risk of cardiac complications (RR: 1.44 95% CI 1.17, 1.78 I^2^ = 19% p = 0.0005) and risk of renal failure in diabetics as compared to non-diabetics (RR: 1.32 95% CI 1.04, 1.68 I^2^ = 0% p = 0.02); but no difference in the risk of cerebrovascular (RR: 1.45 95% CI 0.74, 2.85 I^2^ = 47% p = 0.28), pulmonary (RR: 0.94 95% CI 0.73, 1.23 I^2^ = 8% p = 0.67) and thromboembolic complications (RR: 0.81 95% CI 0.56, 1.17 I^2^ = 28% p = 0.26).

**Conclusion:**

Our results indicate that diabetics have an increased risk of mortality as compared to non-diabetics. Scarce data indicates that the risk of cardiac complications and renal failure are increased in patients with DM but there is no difference in the risk of cerebrovascular, pulmonary, or thromboembolic complications. Further studies are needed to strengthen the current conclusions.

## Introduction

Hip fracture is a debilitating condition that has a high prevalence worldwide. Global estimates suggest that around 4.5 million adults are diagnosed with hip fractures every year resulting in an annual healthcare expenditure of about $9.8 million in the USA alone [1]. Owing to the high fragility of bone and increased tendency of falls, the elderly constitute a significant proportion of patients sustaining hip fractures [2]. Management of such patients entails taking into account several other comorbidities which can impact post-operative recovery and long-term patient outcomes [3].

One of the most common comorbidities found in the elderly is diabetes mellitus (DM). Indeed, DM has been regarded as a global epidemic affecting a large number of patients worldwide. Research indicates that the incidence of DM is on the rise and around 592 million people will be affected by the disease in 2035 [4]. Regardless of the progress in therapeutics and management of DM, diabetes-related complications continue to be a major healthcare problem [5]. Studies indicate that DM has an adverse impact on the skeletal structure resulting in an increased risk of osteoporosis as well as fractures [6, 7]. High glycemic levels associated with DM also lead to osteoblast apoptosis and osteoclast‐mediated bone resorption leading to impaired bone healing and reduced regenerative capacity following trauma [8, 9]. In this context, it is important to understand the impact of DM on outcomes of hip fracture patients.

Diabetic patients are known to have worse functional outcomes after hip fracture surgery as compared to non-diabetics [10]. A meta-analysis by Wei et al. [11] has also demonstrated a significantly increased risk of pressure ulcers amongst DM patients sustaining a hip fracture. However, there have been controversial evidence on the risk of mortality and other major systemic complications between DM and non-DM patients with hip fracture. Some studies have reported increased risk of mortality and systemic complications amongst diabetics [12, 13] while others have reported no such difference [14, 15]. Such variability in results could be due to several reasons like underpowered study, bias in reporting, etc. Therefore, to provide clear evidence to clinicians for better management and risk stratification of diabetic hip fracture patients, there is a need for pooled evidence assessing the impact of DM on complications after hip fracture. Since no such review has been conducted to date, the current study was designed to answer the following research questions: (1) Does DM increase the risk of mortality as in patients with hip fractures? and (2) Are diabetic patients at an increased risk of major systemic complications as compared to non-DM patients with hip fractures?

## Material and methods

This systematic review and meta-analysis is presented according to the reporting guidelines of the PRISMA statement (Preferred Reporting Items for Systematic Reviews and Meta-analyses) [16]. The PROSPERO registration number of the study is CRD42021268525.

### Literature search

Two reviewers independently searched the electronic databases of PubMed, Embase, and Google Scholar for relevant articles. The search strategy was formalized with the aid of a medical librarian and the search limits were set from 1st January 2000 to 1st August 2021. Only English-language studies were included. The search strings used for the literature search were: (1) "diabetes mellitus" AND "hip fractures" and (2) "diabetes mellitus" AND " femoral neck fractures". The same search strings were used for all databases. The primary search results were assessed initially by their titles and abstracts to identify citations requiring full-text analysis. The full texts of the articles were reviewed by the two reviewers independently based on the inclusion and exclusion criteria. Any disagreements were resolved by discussion. We also carried out manual scoping of the bibliography in included studies for any additional articles.

### Inclusion criteria

The inclusion criteria were as follows: (1) All types of studies on adult patients with hip fractures. (2) Studies dividing the sample into two groups of DM and non-DM. (3) Studies comparing mortality or other major systemic complications (cardiac, cerebrovascular, pulmonary, thromboembolic, or renal).

Exclusion criteria were: (1) Non-comparative studies. (2) Studies dividing the sample size based on glucose levels and not on overt DM. (3) Studies assessing only the risk of hip fracture with DM. (4) Abstracts, editorials, review articles, and case reports. (5) Studies with the unavailability of full texts. If more than one study extracted their sample from the same database, the study including the maximum number of patients was selected for inclusion.

### Data extraction and Risk of bias assessment

A data extraction sheet was used by two reviewers to extract relevant data from the studies. Details of the first author, publication year, study type, study location, the database used, sample size, age and gender details, Charlson comorbidity index, percentage of smokers, surgical intervention, maximum follow-up, and study outcomes were extracted.

In the protocol stage we had envisaged to compare “surgical complications, readmission rate, length of hospital stay, and mortality” between diabetic and non-diabetic patients. However, this had to be modified during the conduct of the review due to limited data from included studies on “surgical complications, readmission rate, length of hospital stay”. Instead, majority of the studies reported systemic complications. Hence, the primary outcome of interest for our review was mortality. Secondary outcomes were cardiac, cerebrovascular, pulmonary, thromboembolic, and renal complications. No prior definition was set for the secondary outcomes and all complications related to the particular system were included in the analysis.

The methodological quality of studies was assessed using the Newcastle–Ottawa scale (NOS) [17]. It was conducted by two authors independent of each other. Any disagreements were solved by a discussion. Studies were assessed for selection of study population, comparability, and outcomes, with each domain being awarded a maximum of four, two, and three points respectively. The maximum score which can be awarded was nine. Studies with nine points were considered to have a low risk of bias, seven to eight points were considered to have a moderate risk of bias and those with scores of six and below were with a high risk of bias.

### Statistical analysis

The software "Review Manager" (RevMan, version 5.3; Nordic Cochrane Centre [Cochrane Collaboration], Copenhagen, Denmark; 2014) was used for the meta-analysis. All dichotomous data were summarized using risk ratios (RR) and 95% confidence intervals (CI). We also extracted multivariable-adjusted hazard ratios, odds ratios, or RR for mortality where available. These were then pooled using the generic inverse variance function of the meta-analysis software. Due to limited data, hazard and odds ratios were treated as RR. The random-effects model was used for all the meta-analyses. Heterogeneity was assessed using the I^2^ statistic. I^2^ values of 25–50% represented low, values of 50–75% medium, and more than 75% represented substantial heterogeneity. Due to a limited number of studies in the meta-analysis (less than 10), funnel plots were not used to assess publication bias. We conducted a sensitivity analysis for the meta-analysis on mortality. In the analysis, individual studies were excluded one at a time and the effect size was recalculated for the remaining studies in the meta-analysis software itself.

## Results

### Search results and details of included studies

**Fig. 1:**
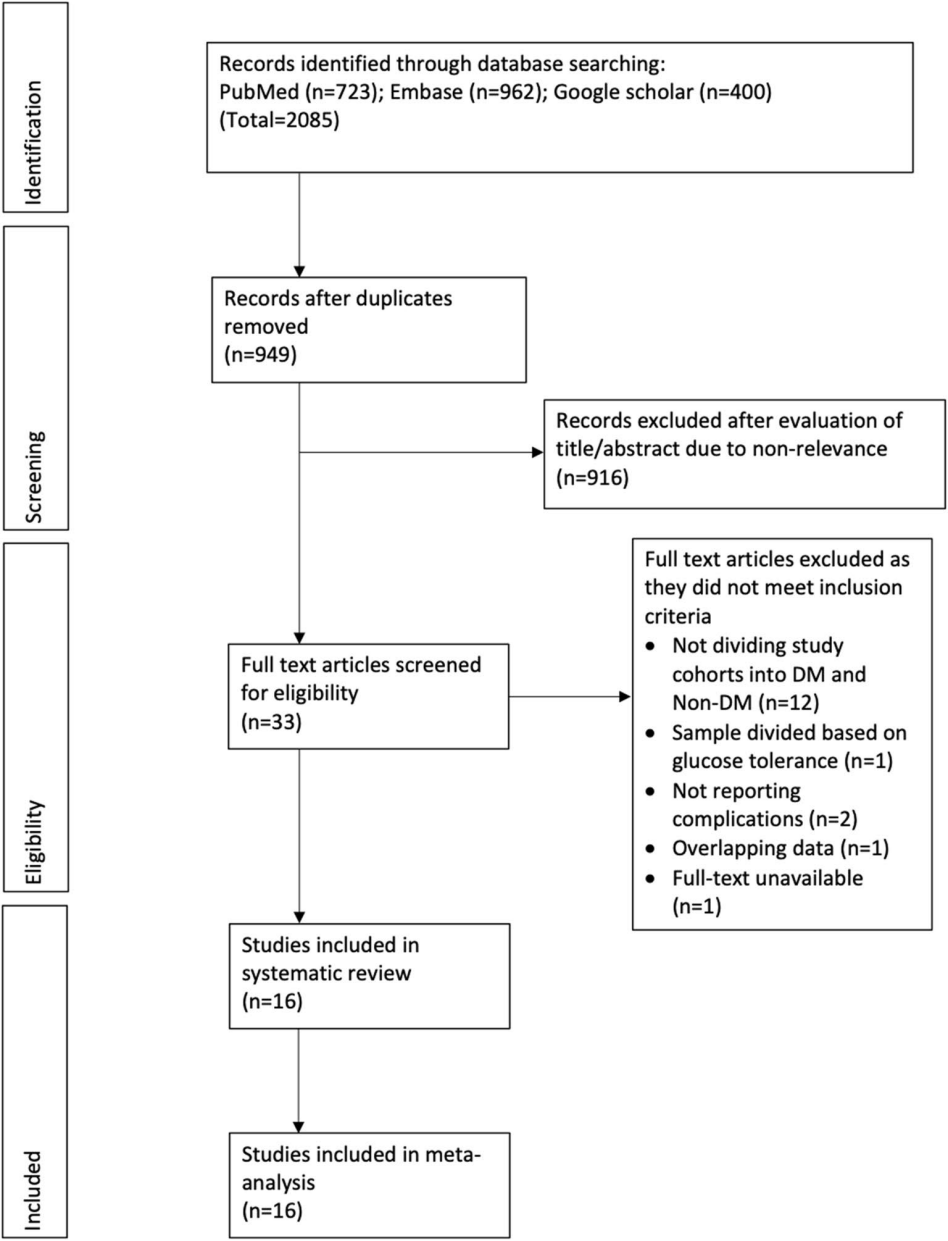
Study flow chart

The number of search results at each stage is summarized in Fig. 1. Of the 949 unique articles searched, 916 were excluded based on title and abstract evaluation. Thirty-three studies were selected for full-text analysis. Seventeen studies were excluded with reasons and a total of 16 studies were included in this review [12–15, 18–29]. Details of included studies are presented in Table 1.

**Table 1:** Characteristics of included studies

Study	Location	Database	Study type	Groups	Sample size	Mean age (years)	Male gender (%)	CCI	Smokers (%)	Surgical treatment (%)	Follow-up
Behanova 2021 [12]	Austria	National database	R	DM Non-DM	8832 45,160	NR	30.6 72	NR	NR	NR	1 year
Rutenberg 2021 [13]	Israel	Single center	R	DM Non-DM	408 935	81 82.9	35 31.9	7[5–9]^ 5[4–7]	8.7 8.2	100 100	Up to 2 years
Tian 2020 [23]	China	Single center	R	DM Non-DM	161 483	77.3 78	27.4 35.2	0.9 ± 1.2 0.7 ± 1	8.1 16.8	80.7 78.1	1 year
Lee 2020 [22]	Taiwan	Insurance database	R	DM Non-DM	2745 2745	80.1 80.5	41.2 41.3	> 2: 41.7% > 2: 41.7%	NR	100 100	1 year
Madsen 2019 [21]	Denmark	National database	R	DM Non-DM	12,158 141,889	79 81	36 30	> 2: 43% > 2: 28%	NR	NR	3 years
Galbraith 2019 [20]	Ireland	Single center	R	DM Non-DM	94 556	79.9 80.3	19.6 20.6	NR	NR	100 100	Up to 2 years
Chandran 2018 [19]	Singapore	Single center	R	DM Non-DM	132 257	76 77	12.9 17.9	4[3–5]^ 4[3–5]	NR	100 100	1 year
Martinez-Laguna 2017 [18]	Spain	National database	R	DM Non-DM	3816 6616	76.6 76.6	24.6 27.1	NR	5.4 4.8	NR	Up to 7 years
Lopez-de-Andrés 2016 [29]	Spain	National database	R	DM Non-DM	92,182 340,578	82.1 83.5	22.6 76.5	NR	NR	95.6 95.2	Up to 10 years
Golinvaux 2015 [14]	USA	National database	R	DM Non-DM	1730 8208	NR	32.9 26	NR	7.8 8	100 100	30 days
Ekstrom 2013 [27]	Sweden	Single center	P	DM Non-DM	234 1899	81.6 81.2	30.5 27.1	NR	NR	100 100	2 years
Wang 2013 [28]	China	Single center	R	DM Non-DM	167 540	72.9 72.1	31.7 33	NR	NR	87.4 91.9	1 year
Huang 2012 [26]	Taiwan	Single center	R	DM Non-DM	61 181	77.3 79.4	29.5 39.2	NR	NR	100 100	1 year
Gulcelik 2011 [24]	Turkey	Single center	R	DM Non-DM	69 161	75.1 76.5	38.5 39	NR	NR	NR	Up to 5 years
Norris 2011 [25]	UK	Single center	R	DM Non-DM	477 5489	77.4 80	22 21.8	NR	NR	NR	1 year
Liebermann 2007 [15]	Israel	Single center	P	DM Non-DM	224 738	NR	28 23	NR	NR	100 100	NR

CCI: Charlson comorbidity index; NR: not reported; DM: diabetes mellitus; NOS: Newcastle Ottawa scale; R: retrospective; P: prospective

^Median {interquartile range]

The included studies were conducted in various countries around the world and published between 2007 and 2021. Except for two prospective cohort studies [15, 27], all were retrospective cohort in nature. Ten studies reported data from a single-center while others analyzed national or insurance databases. There was much variation in the sample size of the included studies. The number of patients in the DM group ranged from 61 to 92,181 while that of the control group ranged from 161 to 141,889. In five studies, the number of patients undergoing surgical intervention was not clear [12, 18, 21, 24, 25]. All patients were treated surgically in eight studies [13–15, 19, 20, 22, 26, 27] while in the remaining studies the number of patients treated surgically was > 78%. In one study [14] the follow-up was just 30 days. In the remaining studies, it ranged from 1 to 10 years.

### Data analysis

**Fig. 2:**
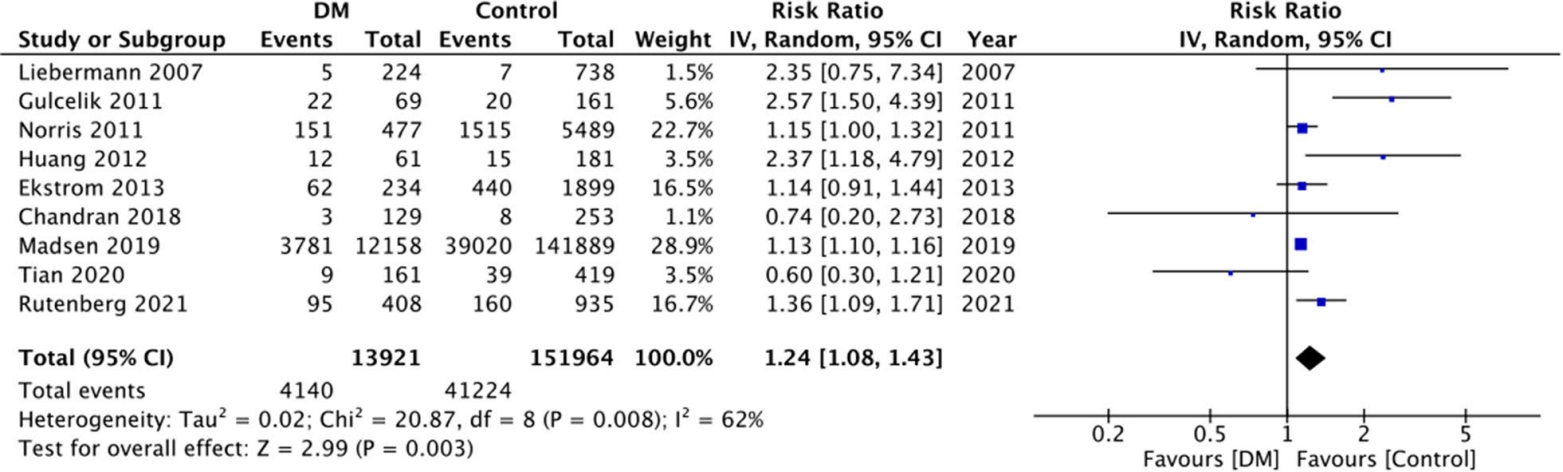
Meta-analysis of crude mortality rates between DM and non-DM patients with hip fractures

**Fig. 3:**
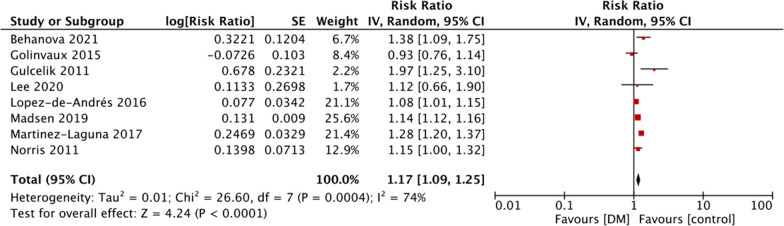
Meta-analysis of adjusted mortality rates between DM and non-DM patients with hip fractures

Mortality data after at least 1 year of follow-up was reported by nine studies. Meta-analysis revealed a statistically significant increased risk of mortality in diabetics as compared to non-diabetics (RR: 1.24 95% CI 1.08, 1.43 I^2^ = 62% p = 0.003) (Fig. 2). Pooled analysis of eight studies reporting adjusted outcomes also demonstrated similar results (RR: 1.17 95% CI 1.09, 1.25 I^2^ = 74% p < 0.0001) (Fig. 3). The results were stable on sensitivity analysis and there was no change in the significance of effect size on the exclusion of any study.

**Fig. 4:**
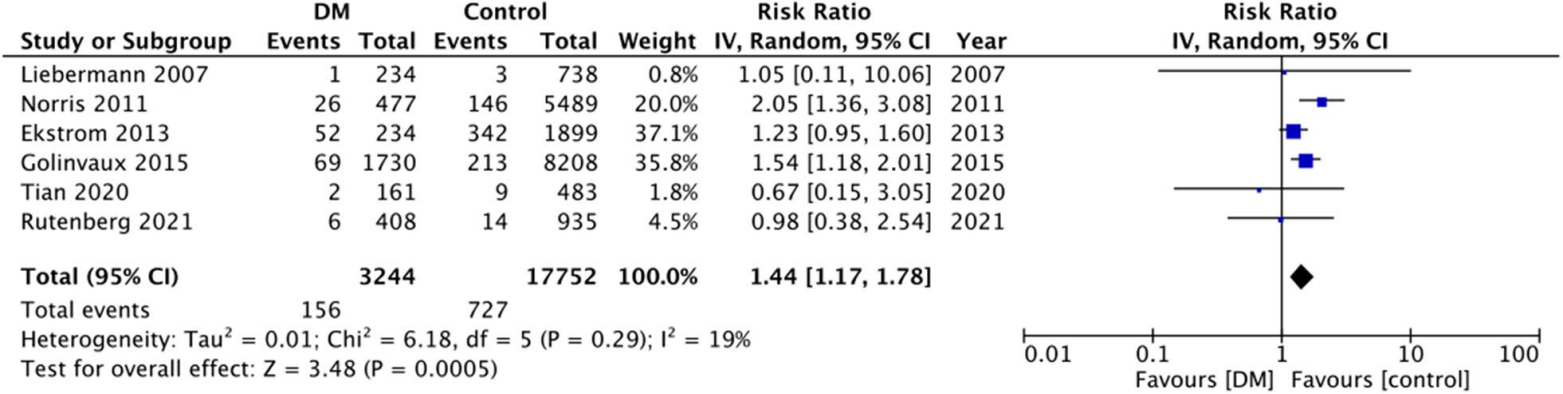
Meta-analysis of cardiac complications between DM and non-DM patients with hip fractures

**Fig. 5:**
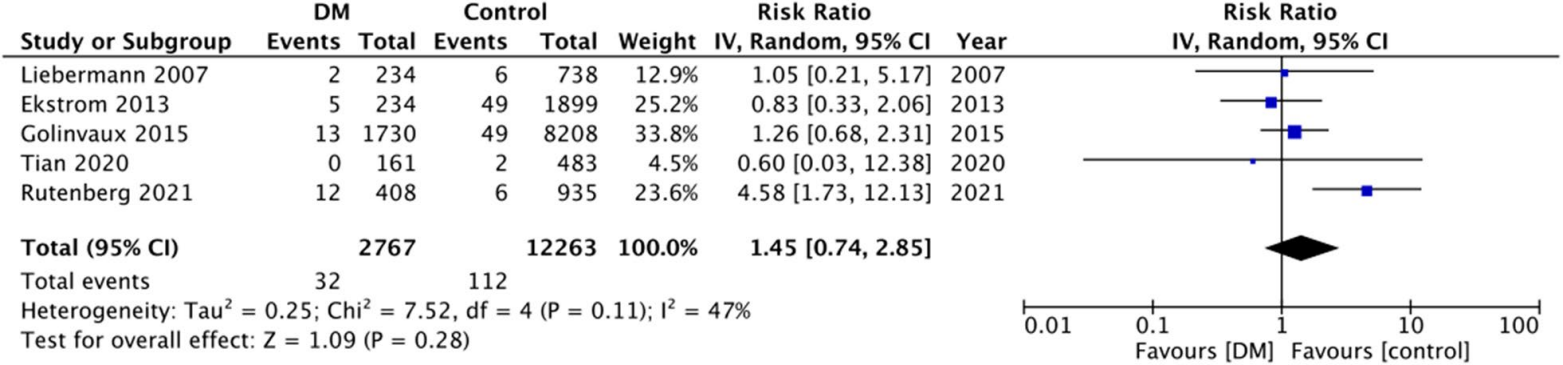
Meta-analysis of cerebrovascular complications between DM and non-DM patients with hip fractures

**Fig. 6:**
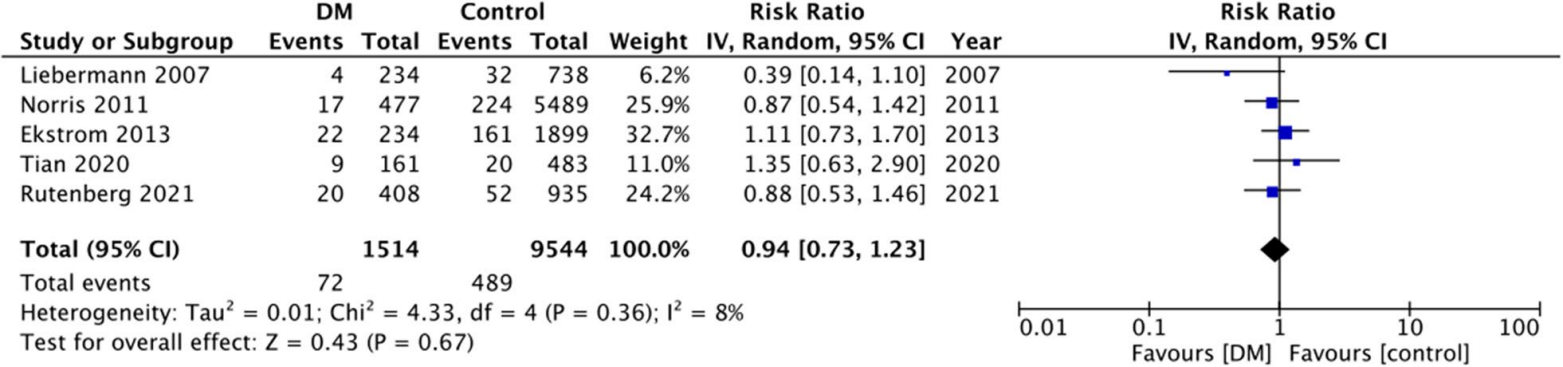
Meta-analysis of pulmonary complications between DM and non-DM patients with hip fractures

**Fig. 7:**
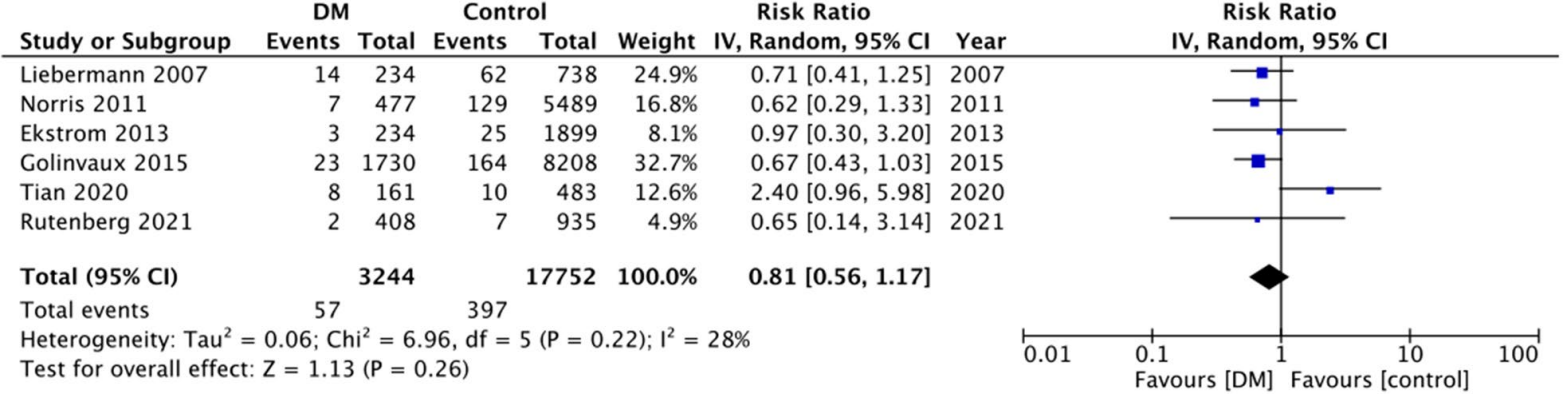
Meta-analysis of thromboembolic complications between DM and non-DM patients with hip fractures

**Fig. 8:**
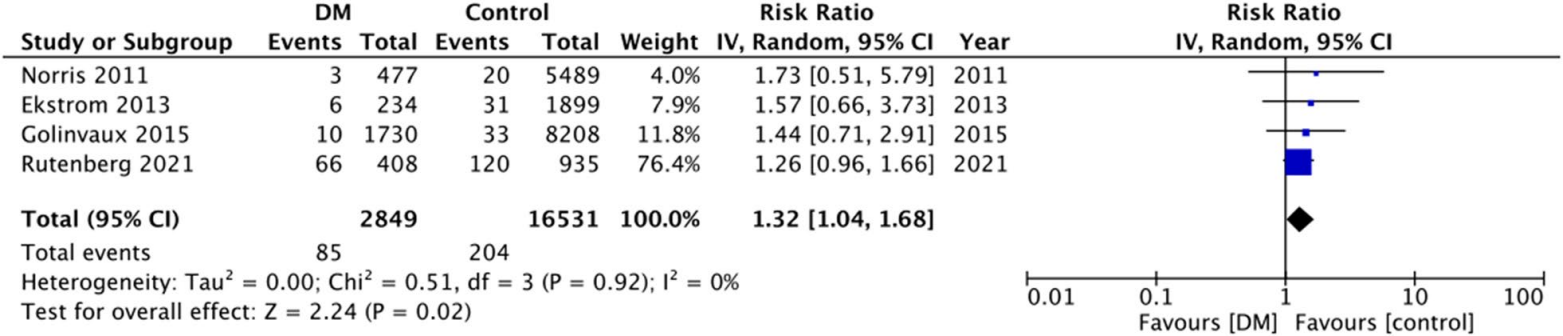
Meta-analysis of renal failure between DM and non-DM patients with hip fractures with hip fractures

For the secondary outcomes, there was variation in the reporting of data by the included studies. A descriptive analysis of outcomes is presented in Table 2. The majority of the studies reported no difference in complications between DM and non-DM groups. However, on quantitative analysis, we noted a statistically significant increase in the risk of cardiac complications in DM patients (RR: 1.44 95% CI 1.17, 1.78 I^2^ = 19% p = 0.0005) (Fig. 4). But on pooled analysis of data from just 5–6 studies, there was no difference in the risk of cerebrovascular (RR: 1.45 95% CI 0.74, 2.85 I^2^ = 47% p = 0.28) (Fig. 5), pulmonary (RR: 0.94 95% CI 0.73, 1.23 I^2^ = 8% p = 0.67) (Fig. 6) and thromboembolic complications (RR: 0.81 95% CI 0.56, 1.17 I^2^ = 28% p = 0.26) (Fig. 7) between DM and non-DM groups. Four studies reported data on renal failure. Meta-analysis revealed a significantly increased risk of renal failure in diabetics as compared to non-diabetics (RR: 1.32 95% CI 1.04, 1.68 I^2^ = 0% p = 0.02) (Fig. 8).

**Table 2:** Descriptive analysis of other outcomes reported by the included studies

Study	Outcome	Results
Rutenberg 2021 [13]	Cerebrovascular event Cardiovascular complications Renal failure Pulmonary complications	Significantly increased in diabetics No difference between two groups No difference between two groups No difference between two groups
Tian 2020 [23]	Cerebrovascular event Cardiovascular complications Pulmonary complications UTI	No difference between two groups No difference between two groups No difference between two groups Significantly increased in diabetics
Lee 2020 [22]	Overall complications^1^	Significantly increased in diabetic patients on insulin but not in patients on oral drugs
Chandran 2018 [19]	Major complications^2^	No difference between two groups
Golinvaux 2015 [14]	Serious adverse event^3^ Non-serious adverse event^4^	No difference between two groups except for myocardial infarction No difference between two groups
Ekstrom 2013 [27]	Cerebrovascular event Cardiovascular complications Renal failure Pulmonary complications Gastrointestinal bleeding Urinary tract infection	No difference between two groups No difference between two groups No difference between two groups No difference between two groups No difference between two groups No difference between two groups
Norris 2011 [25]	Cardiovascular complications Renal failure Pulmonary complications	Significantly increased in diabetics No difference between two groups No difference between two groups
Liebermann 2007 [15]	Cerebrovascular event Cardiovascular complications Pulmonary complications Urinary tract infection	No difference between two groups No difference between two groups No difference between two groups No difference between two groups

^1^Includes acute myocardial infarction, acute stroke, acute renal failure, deep wound infection, pneumonia, postoperative hemorrhagic anemia, septicemia, acute gastrointestinal ulcer, and pulmonary embolism

^2^Includes deep venous thrombosis, pulmonary embolism, cerebrovascular accident, sepsis, urinary tract infection, pneumonia, and arrhythmias that required treatment or acute kidney injury

^3^Includes death, sepsis, septic shock, stroke or cerebrovascular accident, coma, cardiac arrest, myocardial infarction, renal failure, unplanned intubation, ventilator-assisted respiration for greater than 48 h, thromboembolic event, wound-related infection, or return to the operating room

^4^Includes wound dehiscence, renal insufficiency, urinary tract infection, or pneumonia

### Risk of bias

Risk of bias analysis is presented in Table 3. Six studies [12, 15, 23, 26–28] recorded a score of 5–6 on the NOS scale are were deemed to have a high risk of bias. All remaining studies were of moderate risk of bias.

**Table 3:** Risk of bias analysis of included studies

Study	Representativeness	Selection of the non exposed cohort	Ascertainment of exposure	Demonstration that outcome of interest was not present at start of study	Control for confounding factors	Assessment of outcome	Follow up long enough	Adequacy of follow up	NOS score
Behanova 2021 [12]	*	*	*	*	−	*	*	–	6
Rutenberg 2021 [13]	*	*	*	*	**	*	*	–	8
Tian 2020 [23]	*	*	*	*	–	*	*	–	6
Lee 2020 [22]	*	*	*	*	**	*	*	-	8
Madsen 2019 [21]	*	*	*	*	**	*	*	–	8
Galbraith 2019 [20]	*	*	*	*	**	*	*	–	8
Chandran 2018 [19]	*	*	*	*	*	*	*	–	7
Martinez-Laguna 2017 [18]	*	*	*	*	**	*	*	–	8
Lopez-de-Andrés 2016 [23]	*	*	*	*	**	*	*	–	8
Golinvaux 2015 [14]	*	*	*	*	**	*	–	–	7
Ekstrom 2013 [27]	*	*	*	*	-	-	*	–	5
Wang 2013 [28]	*	*	*	*	-	*	*	–	6
Huang 2012 [26]	*	*	*	*	-	*	*	–	6
Gulcelik 2011 [24]	*	*	*	*	**	*	*	–	8
Norris 2011 [25]	*	*	*	*	*	*	*	–	7
Liebermann 2007 [15]	*	*	*	*	–	*	–	–	5

*, single star; **, double star; -, no star

## Discussion

The relationship between DM and fractures has been a subject of intense research in the past decade [6, 7]. Indeed, a recent meta-analysis of 37 studies involving 3,123,382 patients has shown that patients with DM have a 1.5 times increased risk of fracture as compared to non-DM controls. Furthermore, the risk is significantly increased to 5.3 times specifically for hip fractures [7]. While the exact pathophysiological mechanism of this relationship is unclear, research suggests that DM causes alteration in bone quality mediated via insulin-insulin growth factors system, building-up of glycation end-products in bone collagen, microangiopathy, and build-up of bone marrow fat content which increases bone fragility [6]. There are concerns regarding increased osteoclastogenesis, loss of cartilage, decreased tissue mineralization, and altered bone microarchitecture in diabetic patients which may increase the risk of fractures [6, 9]. The advanced glycation end-products accumulate in bone collagen fibers causing alteration in the bone material properties [6]. While insulin has an anabolic effect on bone tissue, it’s long- term use has been linked with an increased fracture risk [6, 9]. Also, diabetics may be at a heightened risk of falls due to associated complications like neuropathy, retinopathy, cognitive impairment, muscle weakness, and hypoglycemia events due to anti-diabetic medications [30]. Thus, the risk of fracture appears to be multifactorial and includes low bone density and bone turnover, altered bone architecture and increased risk of falls due to hypoglycemic risk and other complications. The impact of adverse pathophysiologic events associated with DM is not restricted to just the risk of fracture but also encompasses post-fracture events. Ding et al. [31] in a recent study have indicated that DM patients have a two-fold increase in the risk of impaired fracture healing with lower extremity and short bone fractures being more severely affected by the disease. Considering these factors it may be intuitive to clinicians that DM heightens the risk of post-fracture complications as well, but with contrasting evidence from prior studies and lack of a systematic review and meta-analysis, the true impact has never been quantified.

Since hip fractures are a common type of fragility fracture with generally not-so-satisfactory outcomes [32], additional worsening of the same by DM can be catastrophic. In our analysis of the primary outcome, we noted that DM significantly increased the risk of all-cause mortality after 1-year in hip fracture patients. Quantitatively, there was a 24% increased risk of mortality ranging from 8 to 43%. The results were robust because the difference remained statistically significant even on sensitivity analysis. An important point to note is that several other comorbidities often exist alongside DM which can also contribute to excess mortality. However, even on a pooled analysis of adjusted outcomes from included studies, the results demonstrated a statistically significant 17% increased risk of mortality amongst diabetics. An important drawback of our review was that we were unable to conduct a subgroup analysis on the risk of mortality based on important factors like age, gender, and type of DM due to inadequate data. However, a few studies have segregated outcomes based on these variables. In one of the largest studies, Madsen et al. [21] have reported that excess mortality is more pronounced in patients under 50 years of age as compared to the elderly. Galbraith et al. [20] have noted a significantly higher risk of mortality after hip fracture in male diabetics as compared to female diabetics. Similar results have been reported by Matinez-Laguna et al. [18] in their cohort of any fracture patients. These results concur with the generalized higher risk of mortality noted in men after hip fracture [33, 34]. Considering the type of drug used for DM control, Behanova et al. [12] and Lee et al. [22] have noted significantly higher mortality in hip fracture patients on insulin as compared to those on oral hypoglycemic drugs. Contrastingly, Golinvaux et al. [14] have noted no such difference in outcomes. Excess mortality in insulin-dependent patients may be correlated to the increase in disease severity and prolonged disease duration [12]. However, considering the limited evidence further studies are required to strengthen these results.

In the second part of our analysis, we analyzed other major systemic complications between DM and non-DM patients with hip fractures. However, considering the significant heterogeneity amongst the studies on the type of complications reported, we could include only a limited number of cohorts in the quantitative analysis. In this context, these results should be interpreted with caution. On qualitative analysis of data, it can be noted that the majority of the studies reported no significant difference between DM and non-DM patients for other systemic complications. However, on pooled analysis, we noted a 1.4 timed increased risk of cardiac complications and 1.3 times increased risk of renal failure amongst diabetic patients. The risk of cerebrovascular events, thromboembolic and pulmonary complications were not significantly different amongst the two groups. Similar to our results, several other studies have noted an increased risk of cardiac complications in DM patients undergoing non-cardiac surgery [35–37]. It is well known that DM patients have higher comorbidities and other cardiovascular risk factors than non-DM patients and even the American Heart Association Clinical Practice Guidelines for non-cardiac surgery incorporate DM as an important factor in risk stratification of perioperative cardiac events [38]. The increased risk of cardiac complications has been attributed to increased atherothrombosis, autonomic instability, and heightened systemic inflammation and oxidative stress in diabetics as compared to non-DM patients [35]. Similar to the adverse cardiovascular impact of DM, diabetic nephropathy is a well-known complication of the disease [39]. DM is also an important risk factor of postoperative acute kidney injury which can lead to chronic kidney disease and subsequent renal failure [40]. Despite the increased risk of renal failure noted in our analysis, it should be noted that individually none of the studies reported an increased risk of renal failure in the DM group and the overall increased risk was small with just four studies in the analysis.

The limitations of the current review need to be stated. Foremost, the majority of the studies were retrospective in nature which has inherent selection bias. Furthermore, such studies are prone to bias from errors in data entry and record-keeping, all of which can skew the outcomes of the analysis. Secondly, the total number of studies available for review was not high and there were several studies of small sample size. Inconsistency in data reporting further reduced the number of studies in the meta-analysis. Thirdly, the cause of hip fractures as high-energy or low-energy fractures was not differentiated in some studies and it is possible that some high-energy traumatic injuries were included in the review. However, as the mean age of the patients was > 70 years in most studies, it seems plausible that most patients analyzed in the review were low-energy fragility fractures. Fourthly, our review could assess only if the presence or absence of DM affected outcomes. The relationship between the degree of glycemic control and complications could not be analyzed. Lastly, the type of treatment received by the included patients was not clear in many studies. In only eight studies, 100% of patients received surgical treatment. Due to a lack of data, we could not analyze the difference in complications between surgical and conservatively managed patients.

## Conclusions

The results of the first systematic review and meta-analysis assessing the impact of DM on outcomes of hip fracture demonstrate that diabetics have increased risk mortality as compared to non-diabetics. Scarce data indicates that the risk of cardiac complications and renal failure are increased in patients with DM but there is no difference in the risk of cerebrovascular, pulmonary, or thromboembolic complications. Further studies are needed to strengthen the current conclusions.

## Data Availability

The datasets generated during and/or analysed during the current study are available from the corresponding author on reasonable request.

## Abbreviations

DM: Diabetes mellitus
RR: Risk ratios
